# Primary cilia-associated protein IFT172 in ciliopathies

**DOI:** 10.3389/fcell.2023.1074880

**Published:** 2023-01-17

**Authors:** Nan-Xi Zheng, Ya-Ting Miao, Xi Zhang, Mu-Zhi Huang, Muhammad Jahangir, Shilin Luo, Bing Lang

**Affiliations:** ^1^ Department of Psychiatry, National Clinical Research Centre for Mental Health, The Second Xiangya Hospital, Central South University, Changsha, China; ^2^ Department of Pharmacy, The Second Xiangya Hospital, Central South University, Changsha, China; ^3^ Hunan Provincial Engineering Research Centre of Translational Medicine and Innovative Drug, Changsha, China

**Keywords:** primary cilia, intraflagellar transport, IFT172, ciliopathy, bardet biedl syndrome

## Abstract

Cilium is a highly conserved antenna-like structure protruding from the surface of the cell membrane, which is widely distributed on most mammalian cells. Two types of cilia have been described so far which include motile cilia and immotile cilia and the latter are also known as primary cilia. Dysfunctional primary cilia are commonly associated with a variety of congenital diseases called ciliopathies with multifaceted presentations such as retinopathy, congenital kidney disease, intellectual disability, cancer, polycystic kidney, obesity, Bardet Biedl syndrome (BBS), *etc.* Intraflagellar transport (IFT) is a bi-directional transportation process that helps maintain a balanced flow of proteins or signaling molecules essential for the communication between cilia and cytoplasm. Disrupted IFT contributes to the abnormal structure or function of cilia and frequently promotes the occurrence of ciliopathies. Intraflagellar transport 172 (IFT172) is a newly identified member of IFT proteins closely involved in some rare ciliopathies such as Mainzer-Saldino syndrome (MZSDS) and BBS, though the underpinning causal mechanisms remain largely elusive. In this review, we summarize the key findings on the genetic and protein characteristic of IFT172, as well as its function in intraflagellar transport, to provide comprehensive insights to understand IFT172-related ciliopathies.

## Introduction

Cilium is a highly conserved cell organelle that stands out of the cell surface like an “antenna” and is widely distributed in most eukaryotic cells. Cilia can be grouped into motile and immotile cilia and the latter are also called primary cilia. Every single cell only has one primary cilium that can transduce extracellular information into intracellular signaling cascades to engage in the process of ontogeny and tissue homeostasis (Malicki and Johnson, 2017; Anvarian et al., 2019). Intraflagellar transport (IFT) is a cargo transportation process that moves bidirectionally along the axoneme, the backbone of the cilium (Lechtreck, 2015). IFT depends on two evolutionarily conserved modules, subcomplexes A (IFT-A) and B (IFT-B) to drive ciliary assembly and maintenance. The IFT complexes are assembled at the ciliary base, then move along the ciliary axoneme using the IFT-B machinery to reach the ciliary tip, where they reorganize for retrograde transport to form the IFT-A (Wei et al., 2012). The dysfunction of IFT will result in aberrant structure and function of the cilium and consequently lead to a spectrum of ciliopathies, such as retinal degeneration, congenital heart disease, malignant tumor, polycystic kidney, short ribs-polydactyly syndrome (SRPS), Bardet Biedl syndrome (BBS), Meckel syndrome (MKS) and Joubert syndrome (JS) (Qin et al., 2001; Li et al., 2015; Boubakri et al., 2016; Goetz et al., 2017; Liu and Lechtreck, 2018; Luo et al., 2021; Yang et al., 2021). In addition, defective IFTs also have a close association with neurodevelopmental defects. For example, mutation of *IFT88* could result in neural tube closure defects and brain malformation in mice (Park et al., 2019). As the largest structural component of the IFT-B protein complex, IFT172 is involved in anterograde transportation and thus frequently engaged in diseases including related ciliopathies, growth hormone deficiency, non-syndromic retinitis pigmentosa, MKS, and Mainzer-Saldino syndrome (MZSDS) (Bujakowska et al., 2015; Schaefer et al., 2016; Wang et al., 2018; Pruski et al., 2019). This review mainly focuses on the key findings on the genetic and protein characteristic of IFT172, as well as its function in intraflagellar transport, to provide comprehensive insights to understand IFT172-related ciliopathies.

## Structure of primary cilium

**FIGURE 1:**
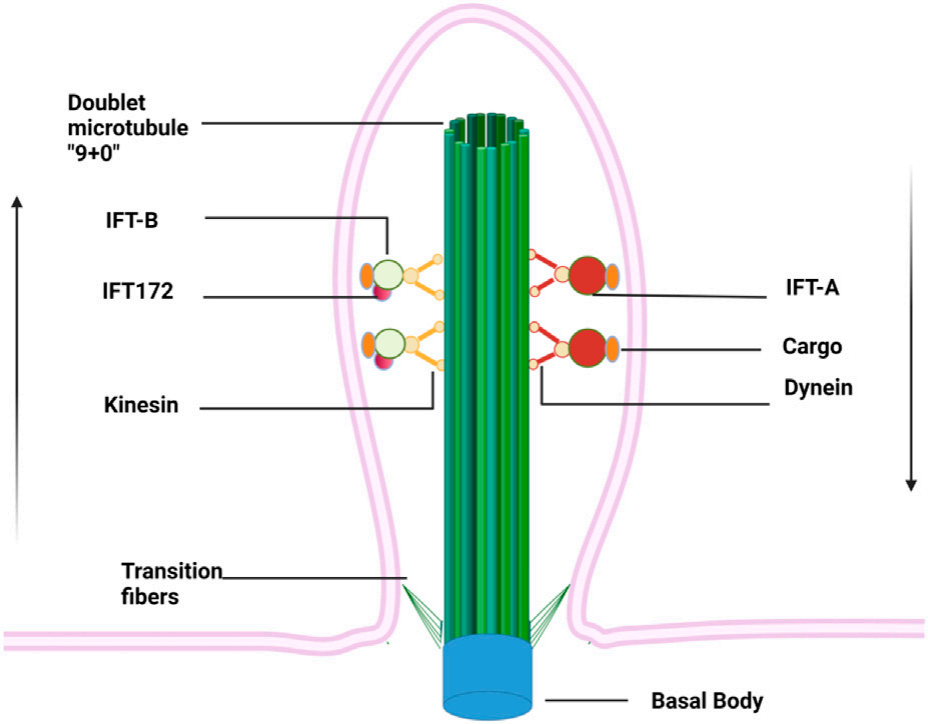
Primary cilium anchors within cytoplasm *via* basal bodies and extends like an “antenna” from a cell surface. Doublet microtubules growing from basal bodies form the backbone of the primary cilium, along which cargos are transported on IFT driven by kinesin and dynein proteins. The IFT complexes are assembled at the ciliary base, then move along the ciliary axoneme using the IFT-B machinery to reach the ciliary tip, where they reorganize for retrograde transport to form the IFT-A. Arrows indicate the direction of cargo flow.

The primary cilium is a thin “hair"-like structure extending from the surface of cell membranes, with a length of about 5–10 μm and a diameter of about 0.2 μm (Yokoyama, 2004). It is present in almost all the post-mitotic cells in mammals but recently its presence in stem cells has also been documented (Bangs et al., 2015). Each cell has only one primary (static) cilium that consists of four indispensable components: the basal body, axoneme, matrix, and ciliary membrane (Figure 1) (Long and Huang, 2019). The basal body is a modified centriole that supports the cilium to anchor with the cytoplasm (Kobayashi and Dynlacht, 2011). The axoneme is composed of nine groups of doublet microtubules that are built upon the basal body and accessory proteins and form the backbone of a cilium. The primary cilium is often regarded as “immotile” due to the absence of the central pair of microtubules (9 + 0) and the inner and outer dynein arms, which serve as “bridges” connecting the nine groups of periphery microtubules. The matrix fills the space between the axoneme and ciliary membrane, which is continuous with the plasma membrane despite unique lipid and receptor composition (Hua and Ferland, 2018). Due to the large surface/volume ratio, more than 600 proteins are enriched within cilium, including many ion channels, neurotransmitters receptors (such as dopamine, serotonin, and somatostatin), platelet-derived growth factor receptors, Hedgehog (Hh) and Wnt signaling pathways receptors (Ishikawa et al., 2012). This feature enables cilia to capture environmental signals sensitively and to trigger intracellular signaling cascades quickly, which consequently regulate gene transcription and protein translation. Interestingly, the length of the cilium is controlled by various kinases and proteins (such as IFT, Tctex-1, and GSK-3β, *etc.*) and thus often associated with the regulation of intracellular signaling pathways as well as changes in the external environment (Avasthi and Marshall, 2012).

## Function of primary cilia

Emerging evidence has strongly suggested that primary cilia play pleiotropic roles, regulating multiple fundamental processes that contribute to the maintenance of cellular activity and tissue homeostases, such as cell autophagy, synaptogenesis, cell division, DNA damage and repair, chromosome separation, and wound healing (Portal et al., 2019). For instance, the elongation of primary cilia and upregulation of associated proteins promote the formation of autophagosomes during the autophagic process of chondrocytes (Ávalos et al., 2017). In the human immune system, neutrophils can sense chemical signals, such as IFN- γ and IL-8, and migrate towards these inflammatory tissues with the primary cilia pointing to the wound. When the primary cilia are defective, this targeted migration may be aborted (Veland et al., 2014).

Primary cilia play an important role in sensory organs responsible for vision, olfaction, and gustation. Cilia connect the inner and outer segments of the rod photoreceptors and the renewal of mammalian visual pigment depends on ciliary transport (Wheway et al., 2014). Additionally, the flow of aqueous humor is perceived by the primary cilia of trabecular meshwork cells (Luu et al., 2018). Olfactory receptors are enriched on the cilia of olfactory epithelial cells (McClintock et al., 2020). Gustation is also achieved through gustatory receptors located on the primary cilia.

During the early stage of vertebrate development, primary cilia control the polarity of somite and thus regulate embryonic development (Sasai and Briscoe, 2012). Dysfunction of primary cilia during the embryonic stage can cause perinatal death in mice with severe craniofacial malformation, cranial neural tube closure defects, holoprosencephaly, cardiac edema, and massive hemorrhage (Vogel et al., 2012; Valente et al., 2014; Fulmer et al., 2019; Portal et al., 2019). During brain development, primary cilia are involved in radial glial scaffold formation and orchestra glutamatergic neuronal migration as well as galvanotaxic migration (Pruski and Lang, 2019). It has been reported that dysfunctional primary cilia may result in corpus callosum agenesis and compromise the functional asymmetry of the brain (Trulioff et al., 2017).

Primary cilia can transmit hypoxia signal to cells and activate an antioxidant signaling cascade and when defective, hypoxia information could not be transmitted into cells. In pulmonary endothelial cells of rats, primary cilia grow significantly longer in a hypoxic environment. Whilst in cultured cells, serum starvation promotes cell cycle exit and initiates ciliary growth (Seeley and Nachury, 2010; Lavagnino et al., 2016). Similarly, Gao *et al.* demonstrated that the primary cilia of PC12 cells may serve as an “oxygen receptor” that can sense the oxidative stress and hypoxia in the external environment and transmit this information to cells to initiate the expression of antioxidant genes such as *HIF-1α* and *Nrf2*(Gao et al., 2017). Intriguingly, a recent study proposed a new kind of synapse, an axon-ciliary synapse, between axons and primary cilia. The primary cilia are enriched in ciliary-restricted serotonin receptor, 5-hydroxytryptamine receptor 6, whose mutation is associated with learning with memory defects. Axo-ciliary synapses are a unique mechanism by which neuromodulators program neurons for transcription through privileged access to nuclear compartments (Sheu et al., 2022).

## Ciliopathies

Defects of primary cilia can lead to many inheritable diseases like polycystic kidney, BBS, JS, MKS, MZSDS, congenital amaurosis, hereditary deafness-pigmented retinitis syndrome (US), SRPS, and asphyxiating thoracic dysplasia (ATD) with overlapping clinical manifestation (Waters and Beales, 2011). BBS patients are often featuring polydactyly, obesity, retinal degeneration, and intellectual problems (Tsang et al., 2018). The majority of MKS patients may die at the perinatal stage due to occipital encephalocele (Hartill et al., 2017). JS patients are characterized by excessive elongation of the superior cerebellar peduncle, deep interphalangeal fossa, and atrophy of the cerebellar vermis together with neurodevelopmental defects such as brain malformation, ataxia, and intellectual impairment (Romani et al., 2013). Intriguingly, these diseases also share some common manifestations. For example, both BBS and JS patients display intellectual disability, hypotonia, and apnea.

Many signaling pathways and receptors are concentrated within cilia, which may play profound roles in the onset of a ciliopathy. The aberrant Hh signaling pathway is demonstrated to underpin the pathogenesis of JS (Aguilar et al., 2012). Similarly, congenital heart diseases are associated with abnormal Hh, Wnt, and Notch signaling pathways that often lead to high comorbidity of ciliopathy (Rohatgi et al., 2007). Recently, abnormal activation of the Hh signaling pathway has been proposed closely related to the occurrence of tumors, such as basal cell carcinoma, breast cancer, and colon adenocarcinoma (Hassounah et al., 2012; Yoshimoto et al., 2012). Active cell division promotes the disassembly of primary cilia, strongly indicating that defects or loss of primary cilia is an important reason for tumorigenesis (Halder et al., 2020).

Impaired primary cilia of neurons are closely associated with intellectual disability, abnormal development of the nervous system, and even mental diseases. G-protein-coupled receptors are enriched in the primary cilia of neurons and regulate the functional maturity of neurons (Mykytyn and Askwith, 2017). Additionally, the defective MCHR1 (a member of GPCRs) is related to the impairment of cognitive function. Type III adenylyl cyclase (AC3), which exists in the primary cilia of neurons, is highly associated with diseases like major depressive disorder, autism, and intellectual disability. Serotonin 5-HT6 receptors are expressed on the surface of the primary cilia of neurons and serve as a potential therapeutic target for Alzheimer’s disease (Hu et al., 2017). Dopamine receptor one localizes to neuronal cilia in a dynamic process that requires the BBS-related proteins (Domire et al., 2011). Importantly, primary cilia regulate neuronal migration and axonal elongation during brain development (Guemez-Gamboa et al., 2014). The impaired primary cilia may lead to agenesis of the corpus callosum, which commonly causes brain asymmetry whilst defective brain asymmetry is frequently associated with mental disorders including autism, dyslexia, depression, bipolar disorder, and schizophrenia (Trulioff et al., 2017). Using the RNA interference technique, Marley *et al.* revealed that 41 widely expressed candidate genes are related to schizophrenia, bipolar disorder, autism spectrum disorder, and intellectual disability. Among these genes, 20 candidates were able to decrease cilium number and three candidates increased cilium length when deleted in NIH3T3 cells, suggesting that different neuropsychiatric risk genes may converge on primary cilia (Marley and von Zastrow, 2012). In the musculoskeletal system, osteocytes and osteoblasts can sense mechanical load *via* primary cilia and contribute to mechanically induced bone formation and maintenance (Moore et al., 2018).

## Components of IFT

Intraflagellar transport (IFT) system was first reported in *Chlamydomonas* as a continuous particle flow powered by dynein (retrogradely) and kinesin (anterogradely) within flagella. Two larger complexes, termed IFT-A and IFT-B (which can be sub-grouped into IFT-B1 and IFT-B2), have been identified containing at least 6 and 16 different IFT particle proteins, respectively (Taschner et al., 2016). Both IFT-A and IFT-B form IFT complexes, which are linked to the axoneme through motor proteins to take cargo running along the ciliary axoneme (Prevo et al., 2017). Moreover, the transition zone at the base of primary cilia is covered by a septin diffusion layer that prevents undesired molecules from entering the cilium, which helps maintain the microenvironment inside the cilium relatively stable and independent of cell solute (Hu et al., 2010).

## Transportation function of IFT

IFT transport is a highly conserved bi-directional flow within the cilium of eukaryotic cells, which is responsible for the transportation of a variety of “cargo”, such as tubulin proteins and some receptor molecules. Apart from this, IFT also has profound roles in the structural assembly and maintenance of primary cilia.

The transport process of IFTs can be roughly described as follows: IFTs are firstly recruited to form the protein complex at the base of the cilium. The anterograde transport is initiated upon the recognition and uploading of the targets of interest. At the tip of the cilia, the IFT complex is autonomously depolymerized and reorganized to activate reverse cargo flow. Finally, IFT components are recycled at the base of cilium for the ongoing cycles of transportation (Chien et al., 2017). Kinesin and IFT-B are involved in the anterograde transport through which proteins essential for cilium assembly could reach the cilium tip for cilium elongation (Ishikawa and Marshall, 2017). Likewise, cytoplasmic motor protein dynein and IFT-A are responsible for retrograde transport. Functional defects in any of the proteins aforementioned will affect cilium formation, length, and normal cargo flow. For instance, dysfunction of IFT-A alters the distribution of neurotransmitter receptors (such as Mchr1, HTR6, and SSTR3) on the ciliary membrane, highlighting the critical role of IFT in maintaining the functional integrity of primary cilia (Picariello et al., 2019).

## Structure of IFT172 gene and protein

**FIGURE 2:**
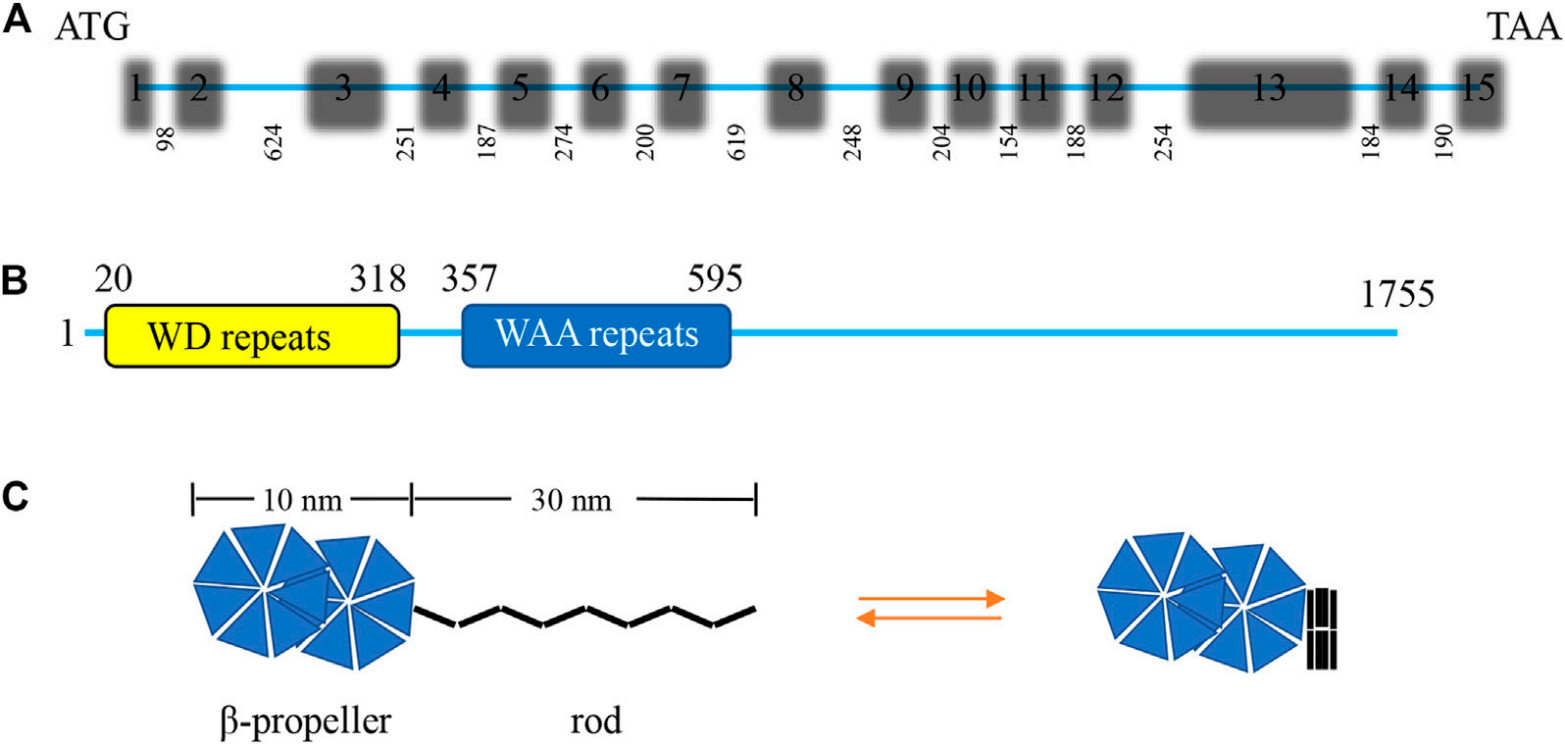
Characterization of the *Chlamydomonas IFT172* gene and its protein. **(A)** The *Chlamydomonas IFT172* gene has 15 exons and 14 introns, the length in bp is listed for each of the introns. **(B)** The domain architecture of IFT172 from *Chlamydomonas*. IFT172 contains seven WD repeats and seven WAA repeats. **(C)** Schematic representation of the conformational transformation of monomeric IFT172.

IFT172 is the largest IFT protein ever discovered, with a molecular weight of about 200 kDa (Wang et al., 2018). It is originally known as a selective LIM domain binding protein (a selective Lhx3/4 LIM-homeodomain transcription factor binding protein, SLB) that participates in the assembly of IFT-B2 (Tsao and Gorovsky, 2008). IFT172 was identified as the 20th component of the BBS complex in 2016 (Schaefer et al., 2016). The *IFT172* gene of *Chlamydomonas* consists of 15 exons and the length of *IFT172* cDNA is 6,091 bp with an open reading frame of 5,265 bp that encodes a protein of 1755 amino acids with a molecular weight of 197.6 kDa, and an isoelectric point of 5.82 (Figure 2A). The amino terminus of the IFT172 protein contains seven WD repeats that are usually involved in transient protein interaction. IFT172 also contains seven distinct degenerate repeats that are called WAA repeats due to the conserved tryptophan and alanine residues (Figure 2B). This sequence is homologous with tetratricopeptide repeats (TPR) and is predicted to present an α-helix secondary structure. The structure of the IFT172 protein is highly conserved in organisms with cilia, and its homologous region includes the WD and WAA domains as described above (Pedersen et al., 2005). IFT172 protein has distinct open and closed conformations, which can be regulated by lipids or detergents. The open conformation consists of two spherical domains of about 10 nm in size with a long rod-shaped protrusion of about 30 nm in length, and the closed conformation features a closed square structure (Figure 2C) (Wang et al., 2018). Through conformational transformation from a slender “rod” shape to a closed “ball” shape, IFT172 assists the anterograde transport of IFT together with “anterograde to retrograde” transition at the ciliary tip possibly through the interaction with *Chlamydomonas* microtubule end binding protein one CrEB1 (Pedersen et al., 2005).

## Function of IFT172

IFT172 is a membrane-associated protein that directly interacts with and remodels the cell membrane. The purified IFT172 has two spherical domains with a long rod protrusion (open conformation), which is similar to the structure of clathrin or COPI-II(Wang et al., 2018). Clathrin mediates cell endocytosis in which substances enter cells by vesicle wrapped with clathrin protein shell *via* protein-protein or protein-lipid interaction (McMahon and Boucrot, 2011), whilst COPI-II mediates vesicle transportation from the endoplasmic reticulum to Golgi (Aridor et al., 1995). Similarly, IFT172 could fuse with lipids, especially those negatively charged, or bind to the lipid membrane through the *β*-helical region of the N-terminal globular domain which can further remodel the membrane into small vesicles of about 20 nm in size. Therefore, IFT172 is a modulatory cargo adapter on IFT, which may first anchor on the primary ciliary base and then recruit other components of IFT to complete assembly (Wang et al., 2018). Besides, other studies have shown that IFT57, one of the essential components of IFT-B2, can compete with the membrane to bind IFT172 through its calponin homology (CH) domain, which subsequently prevents the IFT complex from degradation (Ren et al., 2019).

IFT172 plays an important role in the growth and extension of primary cilia. Quantitative analysis suggested that the length maintenance of primary cilia needs the proper expression of IFT172. Once the expression of IFT172 decreases, the primary cilia will stop growing or even be disassemble, while the total expression of acetylated α-tubulin (the core component of ciliary axoneme) is unchanged (Ma et al., 2020; Yanardag and Pugacheva, 2021). This suggests that the defective growth of primary cilia is a direct consequence of the *IFT172* mutation. IFT172 is necessary for the normal activation of the Hh signaling pathway. In the absence of IFT172, cells can neither promote the formation of Gli activators nor regulate the production of Gli3 inhibitors (Huangfu and Anderson, 2005). Meanwhile, neither complete activation of the Hh pathway caused by *Ptch1* deletion nor complete blockage caused by *Smo* loss had any effect on downstream signaling molecules, suggesting that IFT172 is required for targeted activation of the Hh signaling pathway and is essential for the production of correct amounts of Gli3 inhibitors (Matissek and Elsawa, 2020). Because the Hh signaling pathway is shared by many neurodevelopmental or psychiatric diseases, the mutation of *IFT172* is thus presumably involved in these pathological conditions (Lee and Gleeson, 2011; Wei et al., 2012).

IFT172 plays an important part in retinal development. In mouse embryos, the retina degenerates rapidly when *IFT172* was specifically deleted in rod photoreceptors. In addition, these mice presented overtly decreased electroretinogram (ERG) response at the age of 1 month, complete degeneration of the outer nuclear layer (ONL) at the age of 2 months, and mislocalization of the Hh signaling pathway protein Gli1 (Burnett et al., 2017; Gupta et al., 2018). Apart from this, IFT172 deficiency can lead to olfactory impairment. In zebrafish, *IFT172* deletion results in reduced numbers of olfactory neurons and severely shortened cilia, displaying a declined response to bile acid and food odor but a normal reaction to amino acids. In addition, *IFT172* mutation or deletion in zebrafish can induce intracerebral hemorrhage in an autonomous manner, which may be caused by the dysregulated Hh signaling (Eisa-Beygi et al., 2018; Pollock et al., 2020). *IFT172* mutation has been considered a new cause of anosmia and can be used as a novel diagnostic index of ciliopathies (Bergboer et al., 2018).

In mice, the null mutation of *IFT172* was embryonically lethal. Wimple mice carrying homozygous Leu1564Pro mutation in *IFT172* exhibited developmental stall at embryonic day 10.5–11.5 (E10.5-11.5) with neural tube defects, brain malformations, and preaxial polydactyly (Pruski et al., 2019). Besides, SLB mutant (IFT172^−/−^) embryos died between E12.5–13.5 due to severe craniofacial malformation, incomplete closure of cranial neural tube, malformed forebrain (with no fissure), cardiac edema, and massive hemorrhage (Yuan and Yang, 2015). IFT172 is necessary for the normal function of the embryonic node, early embryonic organizer, and the formation of the head organizing center (the anterior mesendoderm, AME) because of its indispensable roles in cilia morphogenesis and cilia-mediated signal transduction (Gorivodsky et al., 2009). Meanwhile, IFT172 regulates the transition of anterograde and retrograde transportation of IFT at the ciliary tip possibly through CrEB1, as well as participates in the entry or preservation of proteins associated with the ciliary membrane (Pedersen et al., 2005).

## IFT172-related human diseases

In humans, *IFT172* gene mutation frequently results in skeletal ciliopathies, including JS, MZSDS, and Sensenbrenner syndrome with multifaceted manifestations (Halbritter et al., 2013). Halbritter *et al.* sequenced all IFT-B coding genes of 1,467 patients with renal nodular ciliopathies and the whole genome of 63 patients with JS. Twelve families with double allele mutation of *IFT172* were detected, and all affected individuals had abnormalities in the chest and/or long bones, as well as kidney, liver, and retina, consistent with the diagnosis of JS or MZSDS. In addition, two of these 12 families had congenital atrophy or hypoplasia of the cerebellum, characterized by JS (Halbritter et al., 2013). Additionally, the *IFT172* gene mutation leads to retinal degeneration in humans. Bujakowska *et al.* reported four subjects from three *IFT172* mutation families with non-syndromic retinitis pigmentosa, also known as rod-cone dystrophy (Bujakowska et al., 2015). Besides, *IFT172* gene mutation is associated with growth hormone deficiency (Lucas-Herald et al., 2015). Meanwhile, although the causal link between IFT172 and neuropsychiatric diseases still needs to be warranted in future studies, it has been proposed that IFT172 contributes to various neuropsychiatric diseases due to the critical role of IFT172 in embryonic brain development (Youn and Han, 2018; Pruski et al., 2019). In terms of mechanism exploration, although there are no reports of abnormal direct interactions between IFT172 and receptors enriched on primary cilia leading to ciliopathies, in the case of absence of IFT172, these receptors and their coupled downstream signaling pathways are often affected, as cilia are the physical basis for the functions of these receptors, and the absence of IFT172 affects cilia formation (Pala et al., 2017).

## Conclusion and perspectives

Although IFT172 was first reported by Huangfu and his colleagues in 2003, its precise role has not been fully revealed. Recent studies have suggested that IFT172 has membrane binding and remodeling functions, which may be important for IFT-facilitated cargo transportation. IFT172 is also essential for targeted activation of the Hh signaling pathway that closely relates to many human diseases. As a newly identified component of BBS proteins, IFT172 has a fundamental role in the formation and maintenance of primary cilia. Dysfunctional IFT172 promotes the occurrence of many rare ciliopathies with multifaceted manifestations. It should be noted that the involvement of IFT172 in ciliopathies may be underestimated, as the precise diagnosis can only be made after exon sequencing. Therefore, it should be warranted to fully dissect the function of IFT172 in ciliopathies and its underpinning mechanisms to shed novel insights into the diagnosis and treatment of human ciliopathies.
